# Evaluation of mathematical models for predicting medicine distribution into breastmilk - considering biological heterogeneity

**DOI:** 10.3389/fphar.2024.1507551

**Published:** 2024-11-29

**Authors:** Sumin Heo, Andrew S. Butler, Marina Stamouli Simoncioni, Sam Moult, Maria Malamatari, Essam Kerwash, Susan Cole

**Affiliations:** ^1^ Medicines and Healthcare products Regulatory Agency (MHRA), London, United Kingdom; ^2^ Department of Analytical, Environmental and Forensic Sciences, King’s College London, London, United Kingdom

**Keywords:** lactation, breastfeeding, ionisation, milk composition, modelling, infant exposure

## Abstract

**Introduction:**

A significant proportion of mothers take medication during the breastfeeding period, however knowledge of infant safety during continued breastfeeding is often limited. Breastmilk exhibits significant physiological heterogeneity, with a range of milk fat (creamatocrit), protein and pH values available within the literature. Mathematical models for the prediction of infant exposure are available and these predict that variable milk physiology will significantly affect accumulation of drugs within the breastmilk. These models are typically validated against limited datasets only, and to the best of our knowledge no widescale review has been conducted which accounts for the heterogeneity of breastmilk.

**Methods:**

Observed area under the curve milk-to-plasma (M/P) ratios and physicochemical properties were collected for a diverse range of drugs. The reliability of previously published mathematical models was assessed by varying milk pH and creamatocrit across the physiological range. Subsequently, alternative methods for predicting lipid and protein binding within the milk, and the effect of ionisation and physicochemical properties were investigated.

**Results:**

Existing models mis-predicted >40% of medications (Phase Distribution model), exhibited extreme sensitivity to milk pH (Log-Transformed model) or exhibited limited sensitivity to changes in creamatocrit (LogP_o:w_ model). Alternative methods of predicting distribution into milk lipids moderately improved predictions, however altering the way in which milk protein binding was predicted and the effect of ionisation on this demonstrated little effect. Many drugs were predicted to have a significant range of M/P ratios.

**Discussion:**

These data show that consideration of the biological heterogeneity of breastmilk is important for model development and highlight that increased understanding of the physiological mechanisms underlying distribution within the milk may be essential to continue improving *in silico* methodologies to support infant and maternal health.

## 1 Introduction

Breastmilk is an essential and valuable source of sustenance and immunological protection for infants, providing important nutrients and antibodies that promote strong growth and development (Wedekind and Shenker, 2021; Czosnykowska-Łukacka et al., 2018). Over 50% of women take medication during the breastfeeding period, but data regarding the potential exposure of their nursing infants to maternal medication are typically limited, forcing women to choose between their own health and potential harm to their children (Bérard and Sheehy, 2014; Stultz et al., 2007; Al-Sawalha et al., 2016; Ojara et al., 2023; Wang et al., 2017). Although there is increasing demand and regulatory recommendation of the inclusion of lactating women in clinical trials, there is still much work to be done to support safe medicine use during breastfeeding (FDA, 2019; EMA, 2024).

The inclusion of breastfeeding women in clinical trials does present with ethical issues, and so the development of alternative methods of to support increased understanding of potential infant exposure is desirable (Nauwelaerts et al., 2021). A number of non-clinical methods to assess the passage of maternal medication into the breastmilk are available, including *in vitro* human (Kimura et al., 2006; Andersson et al., 2017) and animal models (Ito et al., 2015; Al-Bataineh et al., 2009), *in vivo* animal models (McNamara et al., 1992; Osz et al., 2017) and *in silico* models for the prediction of milk concentrations (Begg and Atkinson, 1993; Fleishaker et al., 1987; Koshimichi et al., 2011; Nauwelaerts et al., 2023). Differences in transporter expression of cell lines have been noted within the literature and *in vitro* work is also complicated by the use of cell-specific media over natural biological matrices (Nauwelaerts et al., 2021; Qu et al., 2015). Similarly, species-specific differences may limit the use of animal models. Rodent models, in particular, have shown a much higher milk-to-plasma (M/P) ratio for some drugs than has been reported in humans (Ito et al., 2013). More recently, Gottingen Minipigs have shown promise as an *in vivo* model, with a strong correlation between minipig and human M/P ratios being reported [unpublished data from IMI ConcePTION (Annaert, 2024)]. Full reviews of *in vitro* and *in vivo* models have been published previously (Nauwelaerts et al., 2021; Ventrella et al., 2019).

The use of mathematical modelling for the *in silico* prediction of maternal M/P ratios is of interest as it removes the concerns regarding species-specific differences in lactation mechanisms. Such models have been used to successfully predict the M/P ratio for medications including primaquine, theophylline, ondansetron and sotalol (Begg and Atkinson, 1993; Pan et al., 2023; Abduljalil et al., 2022; Job et al., 2022). Quantification of maternal plasma concentrations through clinical study and/or physiologically based pharmacokinetic (PBPK) modelling allows M/P ratios to be translated into milk concentrations and therefore the subsequent prediction of infant exposure to maternal medications through breastfeeding (Abduljalil et al., 2022; Job et al., 2022; Abduljalil et al., 2021; Pansari et al., 2022).

Mathematical models for predicting the drug transfer into milk have been of interest for decades, with Atkinson and Begg, and Fleishaker *et al* separately publishing mathematically identical models over 30 years ago (Begg and Atkinson, 1993; Fleishaker et al., 1987; Atkinson and Begg, 1990). These models quantify a ‘phase distribution’ theory of drug partitioning in which the unbound and unionised fraction of drug in the plasma and the breastmilk exists in equilibrium, and differences in milk and plasma concentrations arise due to differences in protein binding, ionisation and lipid partitioning between the two matrices. Natural variability in milk pH, fat content (creamatocrit) and protein content/binding therefore lead to prediction of variable M/P ratios, assuming these factors are sufficiently considered. This Phase Distribution model is supported by *in vitro* bioanalysis conducted by Atkinson and Begg, in which formulae for the prediction of milk protein binding [*fu*_*m*_ (Atkinson and Begg, 1988a)] and lipid partitioning [*Papp*_*milk*_ (Atkinson and Begg, 1988b)] at pH 7.2 were developed based on the known plasma protein binding (*fu*_*p*_) and octanol-to-water distribution coefficient (LogD) respectively. This formula for prediction of *Papp*_*milk*_ shows a steep relationship between LogD_7.2_ and *Papp*_*milk*_, but was developed using drugs with a LogD_7.2_ of <3 only (See Supplementary Figure S2). Following this initial work, Atkinson and Begg employed linear regression to optimise predictions of acidic and basic drugs, with this ‘Log-Transformed’ model providing better predictions than the initial Phase Distribution model (Begg and Atkinson, 1993; Atkinson and Begg, 1990).

More recently, Abduljalil et al. incorporated these mathematical models into PBPK modelling software using an alternative formulation of the Phase Distribution model in which the lipid partitioning is represented by LogP_o:w_ (LogP_o:w_ model). This results in a significantly reduced predicted M/P ratio for lipophilic drugs but was shown to accurately predict the milk distribution of acetaminophen, alprazolam, caffeine and digoxin (Abduljalil et al., 2021; Zhang et al., 2022). Subsequent work by the same group has successfully used both the Phase Distribution and Log-Transformed model to predict infant exposure to a number of medicines (Pan et al., 2023; Abduljalil et al., 2022; Pansari et al., 2022). Although this LogP_o:w_ model represents a misinterpretation of the original Phase Distribution model, it is a useful comparator to study the way in which drug distribution into the milk lipid is incorporated into the model.

In best practice, use of these models includes a sensitivity analysis which accounts for the physiological variability of breastmilk (Pan et al., 2023; Pansari et al., 2022). This is particularly important because lactation studies do not typically include paired recording of factors which are predicted to affect the M/P ratio, such as pH and creamatocrit (Crt), which itself present challenges for model development. Despite their increasing use, to the best of our knowledge there has been no widescale review of the reliability of the Phase Distribution, Log-Transformed or LogP_o:w_ model accounting for the biological heterogeneity of human breastmilk. Additionally, the formulae for prediction of lipid partitioning and milk protein binding have come under little scrutiny since their conception. As such, the present paper aims to assess the reliability of existing mathematical models for predicting the M/P ratio of a diverse list of medications, and to subsequently interrogate additional factors that may alter prediction reliability.

## 2 Methods

### 2.1 Observed milk-to-plasma ratios

Observed M/P ratios were collected from the literature for a diverse range of drugs. Only those for which an area under the curve (AUC) M/P ratio was available were included for analysis. AUC M/P ratios were identified for 91 drugs. Where multiple publications were available for a single drug, the mean M/P ratio was used, weighted for sample size in each publication. The physicochemical properties of each drug were extracted from publicly available databases.

### 2.2 Milk composition

Where possible, details of the observed milk pH and creamatocrit (Crt) were also extracted from the identified literature (see Supplementary Table S2). Milk pH was recorded in 15 studies, whilst creamatocrit was detailed in just 2 of the publications. Mean (±SD) milk pH, weighted for the number of samples in each study, was 7.12 ± 0.24 and so the pH range 6.88–7.37 was used for initial simulations (see Supplementary Figure S1). As such limited data were available on milk Crt, further literature searches were performed. The Crt range of 3%–12% decided upon for investigations (Allen et al., 1991; Meier et al., 2006; Erickson et al., 2013; Mandel et al., 2005).

Predicted M/P ratios were generated by varying pH in twenty identical increments and Crt in increments of 0.1%, thus for each drug ∼1900 simulations were run to generate a range of predicted M/P ratios accounting for the physiological heterogeneity of breastmilk.

### 2.3 Transport-mediated medications

As the models detailed below do not account for the impact of active transport, initial work was conducted using only medications which are not known substrates of drug transporters present in human mammary cells. Transporters expressed in the breast were identified using existing literature (Nauwelaerts et al., 2021; Ventrella et al., 2019) and these data were cross-referenced with ISTransbase (Peng et al., 2024) in order to categorise medicines into those which are and are not mediated by transporters (see Supplementary Table S3).

ABC (MRP2, MRP4, P-gp, BCRP, MRP1, MRP5), SLC (OCTN1, OCTN2, PEPT1, PEPT2, NTCP2, SVCT2, CNT1, CNT3, MCT1, GLUT1, GLUT2, LAT1, OCT1, OCT3, ENT1, ENT3) and SLCO (OATP1A2, OATP3A1, OATP4A1, OATP2B1) family transporters were investigated but only substrates of BCRP, MDR1, MRP2, MRP2, OCTN1, OCTN2, OCT1, OCT3, OATP1A2, OATP2B1 were identified and excluded from initial analysis. 41 medications were identified which are not mediated by transporters thought to be expressed in the breast.

### 2.4 Published lactation models

Predicted M/P ratios were initially generated using the basic Phase Distribution model, the Log-Transformed version of this model (Begg and Atkinson, 1993; Atkinson and Begg, 1990) and the more recent interpretation which used LogP_o:w_ in place of the predicted *Papp*_*milk*_ (LogP_o:w_ model; (Abduljalil et al., 2021)). The Phase Distribution and LogP_o:w_ model calculations are shown below:

$$M/P\;ratio=\frac{{fu}_{p}\cdot f_{p}^{un}}{{fu}_{m}\cdot f_{m}^{un}\cdot S/W\;ratio}$$ (1)

$${fu}_{m}=\frac{{{fu}_{p}}^{0.448}}{{\left({6.94\times 10}^{-4}\right)}^{0.448}+{{fu}_{p}}^{0.448}}$$ (2)

$$S/W\;ratio=\frac{1}{1+Crt\cdot \left({fu}_{m}\cdot {Papp}_{milk}-1\right)}$$ (3)

Where ${fu}_{x}$ , $f_{x}^{un}$ are the unbound and unionised fraction in the plasma (*p*) and milk (*m*) respectively. Fraction unionised was calculated using Henderson Hasselbach equations. The S/W ratio represents the skim-to-whole milk ratio (i.e., the ratio between the concentration of drug in the [aqueous] phase vs. the [aqueous + lipid] phase). ${Papp}_{milk}$ represents the apparent partition coefficient for milk fat and is calculated in the Phase Distribution model as:

$${Papp}_{milk}=10^{-0.88+1.29\cdot {LogD}_{pH,milk}}$$ (4)

and in the LogP_o:w_ model as:

$${Papp}_{milk}={LogP}_{o:w}$$ (5)

LogP_o:w_ values were collected from the literature, whilst LogD_pH, milk_ was calculated from:

$$D_{pH,milk}=P_{o:w}\cdot f_{m}^{un}$$ (6)

The Log-Transformed model used linear regression to separately optimise predictions for acidic and basic drugs, and predicts:

$$Acidic\;drugs:\ln M/P\;ratio=-0.405+9.36\ln\frac{f_{p}^{un}}{f_{m}^{un}}-0.69\ln{fu}_{p}-1.54\ln K$$ (7)

$$Basic\;drugs:\ln M/P\;ratio=0.02477+2.28\ln\frac{f_{p}^{un}}{f_{m}^{un}}+0.886\ln{fu}_{p}+0.505\ln K$$ (8)

where:

$$K=\frac{\left(1-Crt\right)}{{fu}_{m}}+Crt\cdot {Papp}_{milk}$$ (9)

and ${fu}_{m}$ and *Papp*_*milk*_ are calculated using Equation 2 and Equation 4 respectively.

### 2.5 Modified lactation models - *Papp*_*milk*_

Figure 2 presents alternative models for predicting M/P ratios, generated by using modified formulae to calculate ${Papp}_{milk}$ . These are discussed below and detailed in Supplementary Section S2. The models use the following formulae in conjunction with Equations 1–3:

$$ABI\;model:{Papp}_{milk}=10^{\left(0.4017\cdot {LogD}_{pH,milk}\right)+0.1548\;}$$ (10)

$$MCDB\;model:{Papp}_{milk}=10^{\left(2.162-5.327\right)\cdot \exp\left(-0.1153\cdot LogP\right)+5.327}$$ (11)

$$Bartels-LogP\;model:\mathrm{Log}\left({Papp}_{milk}\right)=-4.653+\frac{7.972}{1+10^{0.1175-0.2849\cdot LogP}}$$ (12)

$$Bartels-LogD\;model:\mathrm{Log}\left({Papp}_{milk}\right)=-4.653+\frac{7.972}{1+10^{0.1175-0.2849\cdot {LogD}_{pH,milk}}}$$ (13)

### 2.6 Modified lactation models–*fu*_*m*_

Figure 3 presents alternative models for predicting M/P ratios, generated by using modified formulae to calculate ${fu}_{m}$ . The formulae investigated have been published previously and are detailed in Supplementary Section S3. The models were used in conjunction with the Bartels-LogD model.

$$Yang\;model:{fu}_{m}=1.033\times {fu}_{p}/\left(0.988+{fu}_{p}\;\right)+0.1017\times \ln\left(PSA\right)$$ (14)

$$Ito/Atkinson/Begg\;model:{fu}_{m}=\left({fu}_{p}\times 0.4956\right)+0.5335$$ (15)

Additionally, the *fu*_*p*_ value used to predict *fu*_*m*_ via the Yang (Yang et al., 2022), Ito (Ito et al., 2013) and Atkinson/Begg (see Equation 2) models was modified to account for ionisation as per Lobell et al. [Supplementary Section S3; (Lobell and Sivarajah, 2003)].

### 2.7 Predicted-to-observed ratio

For all models, model reliability was assessed using predicted-to-observed (P/O) ratios, calculated as:

$$P/O\;ratio=\frac{Predicted\;M/P\;ratio\;}{Observed\;M/P\;ratio}$$ (16)

Drugs were considered as predicting within 2- or 5-fold of the observed data if *any* predicted M/P across the physiological range of milk being investigated was within that range. For example, a drug with a predicted M/P range of 1 – 3 would be considered within 2-fold of the observed data if the observed M/P ratio was between 0.5 and 6.

### 2.8 Software

All predictions were made using RStudio version 4.4.0 (2024-04-24) (Posit Software). Scripts are available upon request.

## 3 Results

### 3.1 Reliability of published lactation models

**FIGURE 1:**
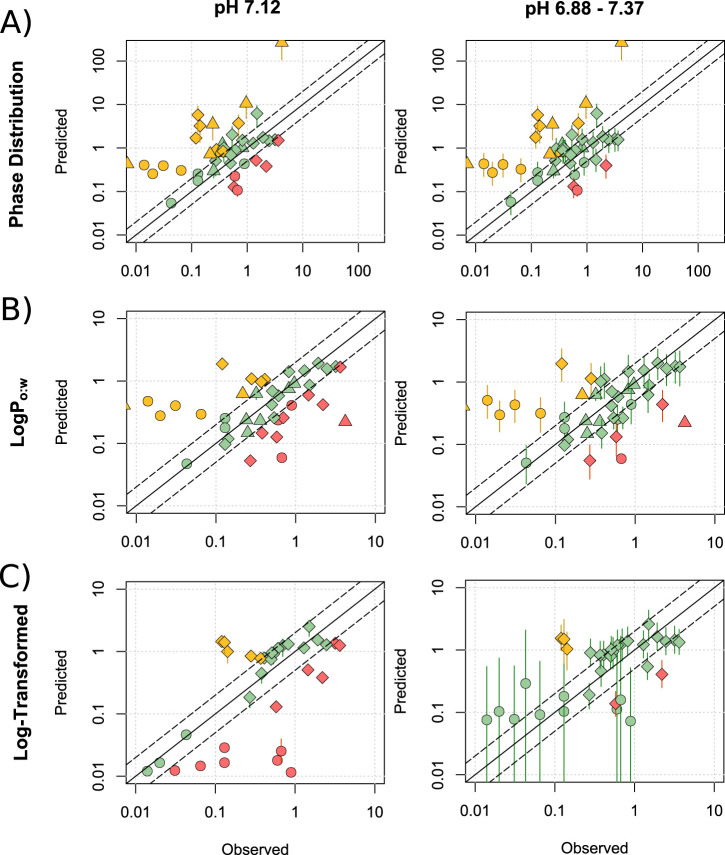
Assessment of published lactation models. Mean (range) predicted M/P ratios versus the observed data for the Phase distribution **(A)**, LogP_o:w_ **(B)** and Log-Transformed **(C)** models, for drugs which are not substrates of transporters expressed within the breast. Predictions were made at pH 7.12 and across the range of 6.88–7.37. Solid and dashed black lines represent a P/O ratio of 1 and within 2-fold respectively. Data points are coloured to highlight over-predictions (yellow), underpredictions (red) and predictions within 2-fold (green), whilst shapes represent acids (○), bases (◇) and neutral drugs (□). Data are considered within 2-fold if any value in the predicted range is within 2-fold of the observed data.

Predictions of M/P ratios were first generated for the Phase Distribution, LogP_o:w_ (Equations 1–6) and Log-Transformed (Equations 7–9) models for 41 drugs (not mediated by transporters) at pH 7.12 (Figure 1, left) and with a Crt range of 3%–12%. At pH 7.12, the Phase Distribution model showed a tendency to overpredict (Figure 1A; 39.0% >2-fold over-predicted; 14.6% underpredicted), whilst the Log-Transformed model tended towards underprediction (Figure 1C; 28.5% underpredicted and 11.9% overpredicted) and the LogP_o:w_ model showed more balance, but a similarly low overall prediction reliability (Figure 1B; 24.4% overpredicted and 26.9% underpredicted). It was also noted that for lipophilic drugs such as zolpidem, the LogP_o:w_ model showed relative insensitivity to changes in Crt (M/P range of 0.09–0.10) compared to the Phase Distribution and Log-Transformed models (ranges of 2.35–9.16 and 0.91–1.80 respectively).

Expanding the milk parameters to pH 6.88–7.37 increased proportion of drugs which were predicted within 2- and 5-fold of the observed data (righthand side of Figure 1). The Phase Distribution model (Figure 1A) predicted 61.0% and 78.0% of drugs within 2- and 5-fold respectively, compared with 68.3% and 82.9% for the LogP_o:w_ model (Figure 1B) and 84.8% and 97% for the Log-Transformed model (Figure 1C). The Log-Transformed model also exhibited a lower mean (±SD) average fold error (AFE) of 3.68 ± 3.24, compare with 8.87 ± 15.08 for the Phase Distribution model and 5.97 ± 10.76 for the LogP_o:w_ model. The mean predicted M/P ratios exhibited limited linear correlations with the observed values (0.29, 0.21 and 0.21 for the Phase Distribution, LogP_o:w_ and Log-Transformed models respectively).

Although the statistics presented above suggest that the Log-Transformed model predicts better than the other models, the predicted range of M/P ratios for acidic drugs was extremely large (>900-fold difference between minimum and maximum predicted M/P ratio), demonstrating the model to exhibit a high sensitivity to changes in pH. This range does not accurately reflect the observed data, and makes the predicted M/P ratios difficult to interpret fully. In addition to poorly predicting acidic drugs, there was little linear correlation between the predicted and observed values for basic drugs using the Log-Transformed model (R^2^ = 0.08). It also does not include a formula for prediction of neutral drugs. These data are summarised in Supplementary Table S4 and all data points listed in Supplementary Tables S5–S8.

### 3.2 Interrogating *Papp*_*milk*_

Given these limitations of the Log-Transformed model, we opted to investigate the Phase Distribution and LogP_o:w_ models. Although the LogP_o:w_ model showed better prediction of drug distribution in the breastmilk, it was insensitive to changes in Crt and appears to be no longer used by the authors, with the Phase Distribution (and Log-Transformed) models being preferred, likely due to a stronger experimental basis (Abduljalil et al., 2022; Abduljalil et al., 2021; Pansari et al., 2022). The incorporation of drug lipophilicity is the only difference between the Phase Distribution and LogP_o:w_ models (see Equations 4, 5), and it is therefore noteworthy that the Phase Distribution model significantly *overpredicted* the M/P ratio of lipophilic drugs: 6/7 drugs with a LogD > 2.3 were overpredicted, with a mean (±SD) P/O ratio of 23.3 (±22.6) see Equation 16. In comparison, 7/14 drugs with a LogP > 2.3 were *underpredicted* by the LogP_o:w_ model, and only 2/14 overpredicted (P/O ratio of 0.94 ± 1.1). Equation 4 was originally derived through the assessment of milk lipid partitioning in a limited dataset only, and shows a steep relationship between LogP_o:w_ and LogPapp_milk_ [See Supplementary Figure S2; (Atkinson and Begg, 1988b)]. As such, we investigated alternative models for predicting *Papp*_*milk*_. These are detailed in Supplementary Section S2 but in brief:

• The ABI model pools the original milk lipid partitioning data with a more modern data set (Ito et al., 2013), with the pooled data being best fit by a linear equation (Equation 10).
• The MCDB model is based on ∼680 datapoints describing the lipid partitioning of ∼150 compounds in bovine milk (Foroutan et al., 2019), with data being best fit by an exponential function (Equation 11).
• The Bartels model relies on published correlations between the LogP (Equation 12) or LogD (Equation 13) and the olive oil partitioning (LogP_vo:w_) coefficient (Bartels et al., 2012).

**FIGURE 2:**
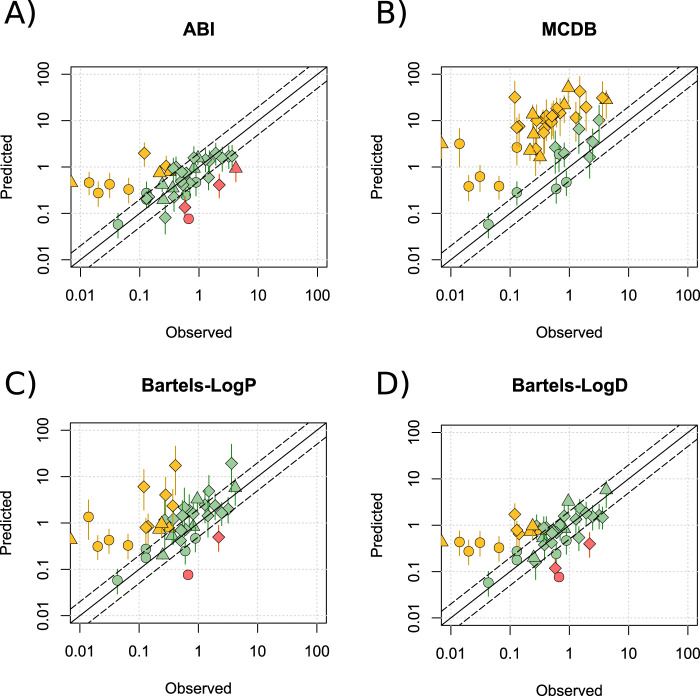
Alternative methods for predicting *Papp*_*milk*_. Mean (range) predicted M/P ratios versus the observed data for the ABI **(A)**, MCDB **(B)**, Bartels-LogP **(C)** and Bartels-LogD **(D)** models, for drugs which are not substrates of transporters expressed within the breast, at a pH of 6.88–7.37 and a Crt of 3%–12%. Solid and dashed black lines represent a P/O ratio of 1 and within 2-fold respectively. Data points are coloured to highlight overpredictions (yellow), underpredictions (red) and predictions within 2-fold (green), whilst shapes represent acids (○), bases (◇) and neutral drugs (□). Data are considered within 2-fold if any value in the predicted range is within 2-fold of the observed data.

Predictions were run across a pH range of 6.88–7.37 and a Crt range of 3%–12%. The ABI and Bartels-LogD models offered a modest improvement over the Phase Distribution model, with 68.3% and 85.4% (ABI; Figure 2A), and 61.0% and 85.4% (Bartels-LogD; Figure 2D) of drugs predicting within 2- and 5-fold, respectively. Thus, these models demonstrated similar reliability to the LogP_o:w_ model, but with a stronger basis in experimental evidence and with a more appropriate response to changes in Crt. A significant improvement in prediction of lipophilic drugs was recorded for both models. A small increase in R^2^ was observed with the ABI model (R^2^ = 0.31) but a stronger association between the observed and predicted values was identified by the Bartels-LogD model (R^2^ = 0.51). A slight reduction in mean (±SD) AFE was also seen for the ABI (5.54 ± 11.17) and Bartels-LogD (5.47 ± 10.51) models compared to the Phase Distribution model. These improvements in prediction were not noted for the MCDB model, which consistently overpredicted M/P ratios (Figure 2B), or for the Bartels-LogP model (Figure 2C). These data are summarised in Supplementary Table S4 and all data points listed in Supplementary Tables S7–S8. As the best performing model, the Bartels-LogD model was used for all simulations in the remainder of the manuscript.

### 3.3 Interrogating *fu*_*m*_

**FIGURE 3:**
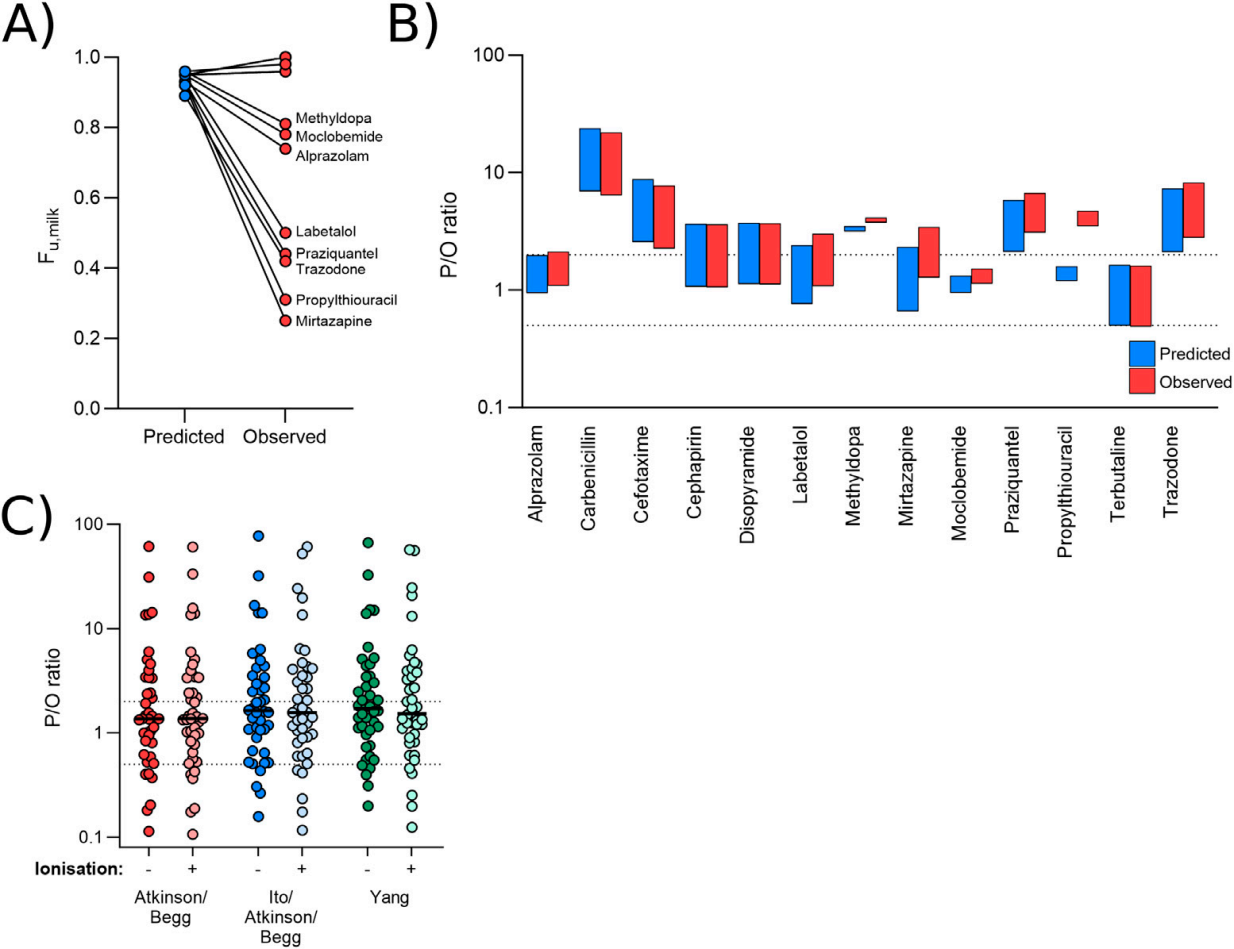
Effect of observed versus predicted protein binding. (A) Paired predicted and observed *fu_m_* values for each drug in the dataset for which an observed value was identified. **(B)** Range of P/O ratios calculated using the Bartels-LogD model and either the predicted (blue) or observed (red) *fu_m_* value. **(C)** Mean P/O ratios calculated for each of the 42 drugs using the Bartels-LogD model in conjunction with the Atkinson/Begg (red), Ito/Atkinson/Begg (blue) or Yang (green) formulae for predicting fraction unbound in the milk, with or without incorporating the effect of ionisation. In **(B, C)**, the dashed lines represent 0.5 and 2 (2-fold error).

In addition to predicted values for *Papp*_*milk*_, the models also depend upon a predicted value for protein binding within the skimmed fraction of the milk (Equation 2). Similar to the above, this prediction is based on a low number of drugs (Atkinson and Begg, 1988a) and it calculates *fu*_*m*_ using *fu*_*p*_ only. For a fraction of drugs analysed above (13/42), an observed *fu*_*m*_ was available and in some instances these were significantly different to the predicted values (Figure 3A). As such, simulations were run using the observed values for *fu*_*m*_ in place of those predicted by Equation 2.

The observed value did not significantly impact predictions for most drugs (Figure 3B). For one drug (propylthiouracil; observed M/P = 0.13), the use of the measured value shifted the range of predicted M/P ratios from 0.16-0.21 to 0.46-0.61, reducing the prediction accuracy ∼3-fold.

In addition to the Atkinson/Begg model for predicting *fu*_*m*_, other methods are available within the literature (Ito et al., 2013; Yang et al., 2022) see Equations 14, 15. Additionally, increased ionisation reduces binding to plasma proteins, and so it is plausible that changes in milk pH may also be associated with a change in *fu*_*m*_. This phenomenon is not currently incorporated into the model, but can be estimated using Lobell and Sivarajah’s (2003) work which drew correlations between the ionisation state of a compound, it’s lipophilicity, and it’s binding to plasma proteins ((Lobell and Sivarajah, 2003); see Supplementary Section S3). The effect of alternative methods for predicting *fu*_*m*_ including or excluding the effect of ionisation were therefore investigated. As shown in Figure 3C, none of these methods significantly affected prediction reliability. The Yang model (minus ionisation), which incorporates polar surface area (PSA) into the prediction of *fu*_*m*_, did increase the predicted M/P ratio for some underpredicted medications. As such, only one medication (phenacetin) was >2-fold underpredicted by this model (overall 69.0% within 2-fold and 88.1% within 5-fold).

### 3.4 Interrogating the effect of transporters

**FIGURE 4:**
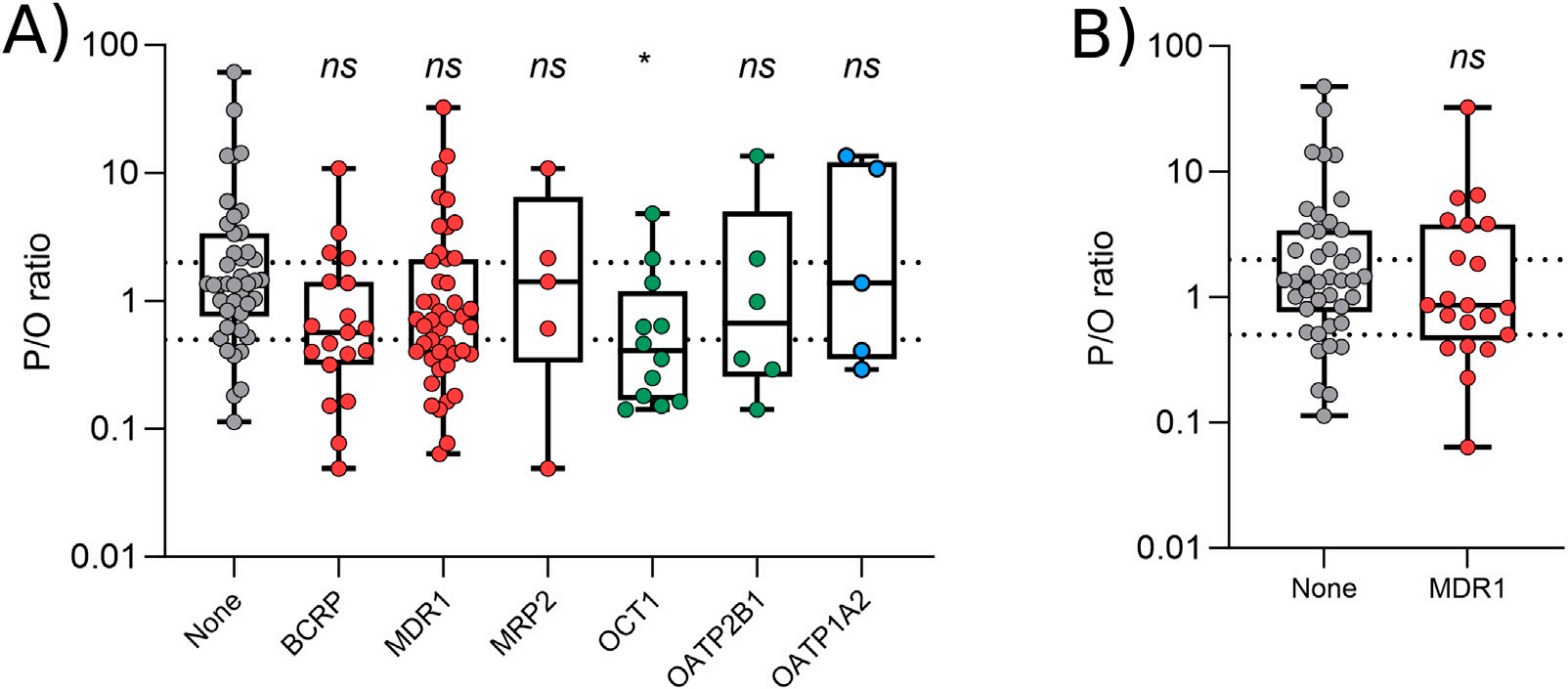
Effect of transporters on prediction reliability. (A) Mean predicted P/O ratios for drugs which are not substrates of transporters expressed in the breast (grey), and for those mediated by apical (red) and basolateral (green) efflux transporters or apical influx transporters (blue). Statistics represent a Kruskal–Wallis ANOVA with Dunn’s multiple comparison, comparing each transporter with the control group. **(B)** Predicted P/O ratios for drugs which are not substrates of transporters expressed in the breast (grey), and for those transported by MDR1 only (red). Statistics represent an unpaired *t*-test. All data were generated using the Bartels-LogD model and the dotted lines represent 0.5 and 2 (2-fold error).

For the simulations presented above, 51 substrates of transporters expressed in the breast were excluded from analysis as it may be expected that active transport would contribute to mispredictions. The impact of 26 transporters was investigated (see Methods), however there were only 6 transporters for which a reasonable number (≥5) of substrates were identified (BCRP, MDR1, MRP2, OCT1, OATP2B1 and OATP1A2). Compared to the drugs which were not mediated by transporters, there was no significant difference in the prediction reliability (P/O ratio) for substrates of BCRP (p = 0.068), MDR1 (p = 0.12), MRP2 (p > 0.99), OATP2B1 (p > 0.99) or OATP1A2 (p > 0.99; Figure 4A). The P/O ratio of OCT1 substrates, however, was significantly lower (p < 0.05; Figure 4A) than drugs not mediated by transporters, suggesting that the M/P ratio for OCT1 substrates is typically underpredicted.

These data are complicated somewhat by the fact that transport of most drugs is mediated by multiple transporters (see Supplementary Table S3 for breakdown). When limiting the transport-mediated drug lists to those which are only mediated by a single transporter, only a significant number of MDR1 substrates were available. The P/O ratio of drugs mediated *only* by MDR1 was also not significantly different from those drugs not mediated by any transporter (Figure 4B).

### 3.5 Interrogating the effect of pH

**FIGURE 5:**
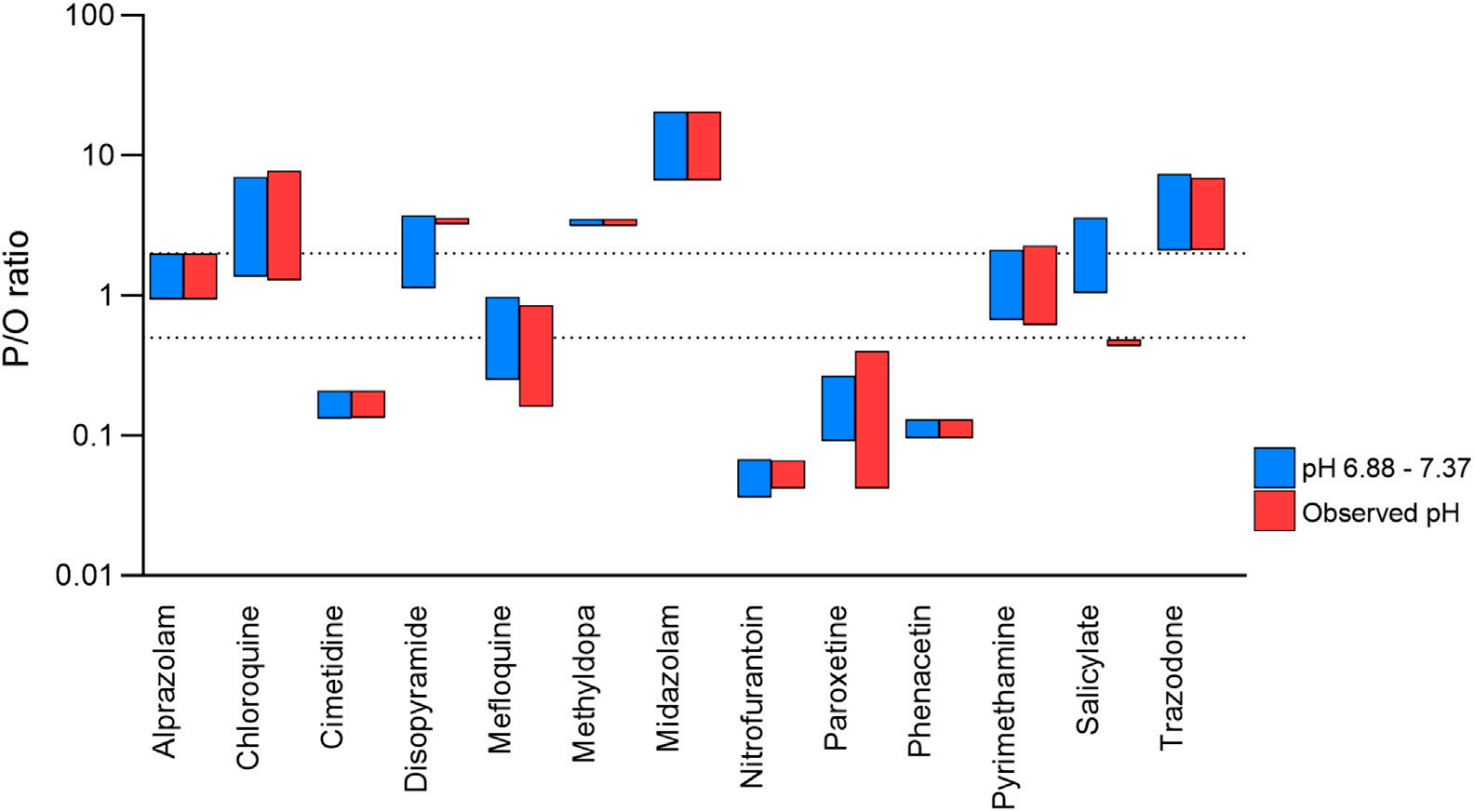
Effect of observed versus mean milk pH values on prediction reliability. Range of P/O ratios calculated using the Bartels-LogD model and either the mean ± SD (blue) or observed (red) milk pH values. The dashed lines represent 0.5 and 2 (2-fold error).

The simulations presented above, all used a pH range of 6.88–7.37. To investigate whether this pH range had a significant effect on predictions, simulations were also run for the 13 drugs for which matched observed pH data were available. Predictions using the observed pH range showed broad agreement with the predictions presented above, although prediction reliability was reduced for two drugs (disopyramide and salicylate; Figure 5).

**FIGURE 6:**
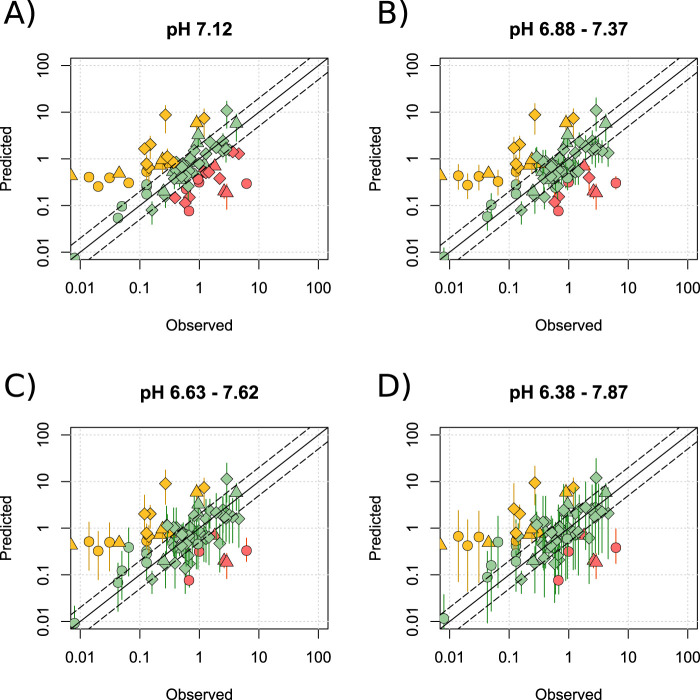
Effect of widening the range of milk pH. Mean (range) predicted M/P ratios versus the observed data for the Bartels-LogD model at a pH of 7.12 **(A)**, 6.88–7.37 **(B)**, 6.63–7.62 **(C)** or 6.38–7.87 **(D)**. BCRP, MDR1 and OATP2B1 substrates were analysed in addition to drugs which are not mediated by transporters expressed within the breast. Solid and dashed black lines represent a P/O ratio of 1 or within 2-fold respectively. Data points are coloured according to highlight overpredictions (yellow), underpredictions (red) and predictions within 2-fold (green), whilst shapes represent acids (○), bases (◇) and neutral drugs (□). Data are considered within 2-fold if any value in the predicted range is within 2-fold of the observed data.

To further investigate the effect of pH, simulations were run at the mean observed pH (7.12) ± 0, 1, 2 and 3 standard deviations (See Supplementary Figure S1). Substrates of BCRP, MDR1, MRP2, OATP2B1 and OATP1A2, were included in this analysis given that these transporters were not shown to affect prediction reliability. Increasing the pH range led to an increased number of predictions matching the observed data, with 53.2% and 82.3% being within 2- and 5-fold, respectively when only pH 7.12 was used (Figure 6A), compared with 73.4% and 88.6% when using the more complete physiological range of pH 6.38–7.87 (Figure 6D). Figure 6D also shows that over the largest pH range investigated, the predicted range of M/P ratios for ∼25% of drugs was large (>30-fold). For example, the M/P ratio of atenolol was predicted to be between 0.3 and 10.2. These data are summarised in Supplementary Table S4 and detailed in Supplementary Table S9.

### 3.6 Interrogating the effect of physicochemical properties

**FIGURE 7:**
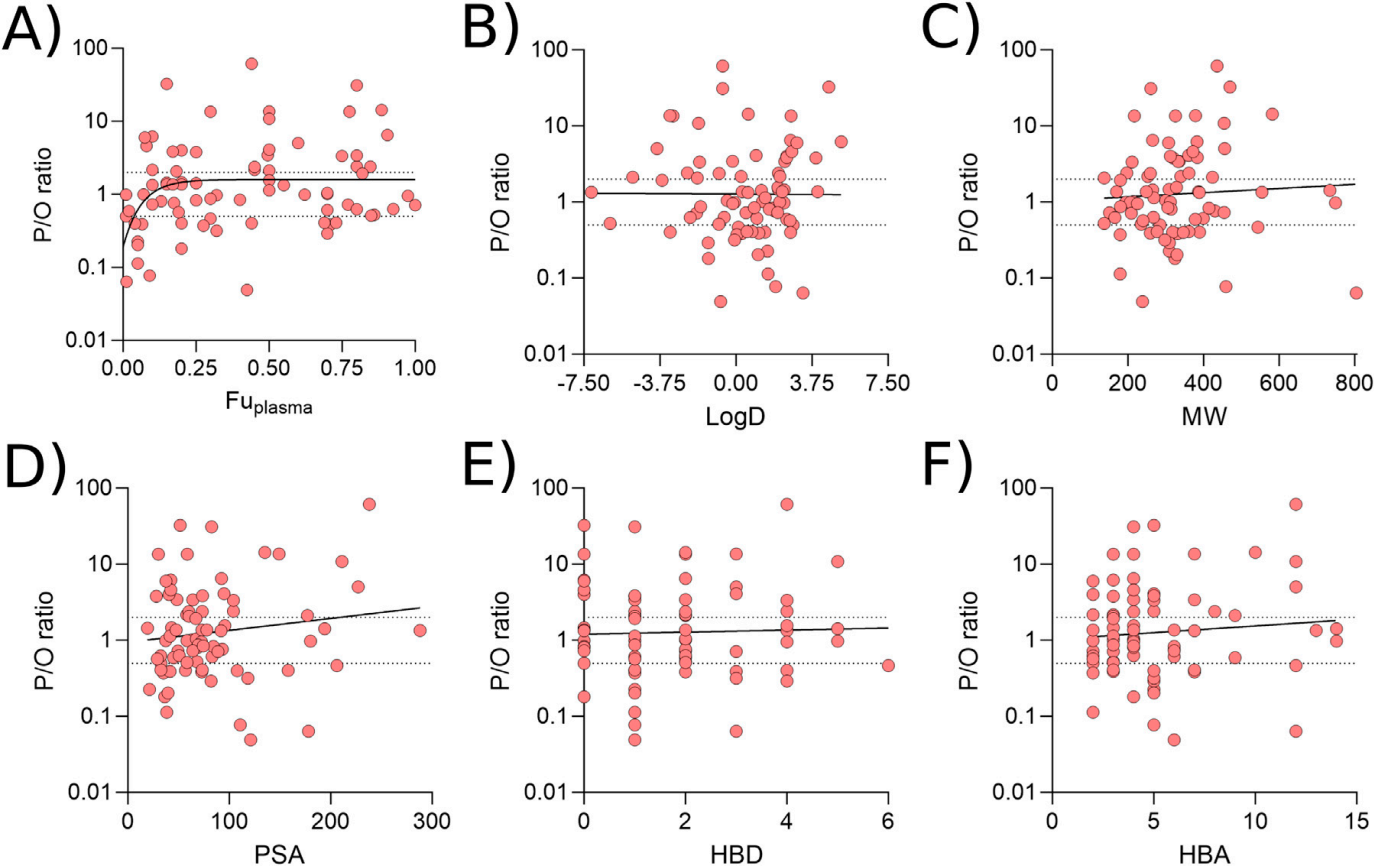
Associations between prediction reliability and physicochemical properties. Correlations between the P/O ratio and Fu_plasma_ **(A)**, LogD **(B)**, molecular weight **(C)**, polar surface are **(D)**, number of hydrogen bond donors **(E)** and number of hydrogen bond acceptors **(F)**. All data were generated using the Bartels-LogD model and drugs which are substrate of transporters expressed in the breast, or are BCRP1, MDR1 or OATP2B1 substrates. All plots were best fit by linear equations other than A.

To investigate whether particular physicochemical properties affected prediction reliability, correlations were drawn between the P/O ratio and *fu*_*p*_, LogD, molecular weight (MW), PSA, number of hydrogen bond donors (HBD) and acceptors (HBA). The relationship between P/O ratio and *fu*_*p*_ was best described by a non-linear relationship suggesting that drugs which exhibit high protein binding in the plasma (*fu*_*p*_ < 0.05) were more likely to be underpredicted (Figure 7A). No relationship between prediction reliability and LogD, MW, PSA, HBD or HBA was identified (Figures 7B–F).

Finally, it was noted that basic dugs predicted better than both acidic or neutral drugs, with 78.6% and 92.9% of basic medicines predicting within 2- and 5-fold, respectively, compared with 57.9% and 73.7% for acids and 50.0% and 77.8% for neutral drugs when predictions were made at pH 6.88–7.37. Increasing the pH range to 6.38–7.87 led to 88.1% of basic drugs to be predicted within 2-fold (95.2% within 5-fold).

## 4 Discussion

All models evaluated showed misprediction of a significant proportion of the drugs. Of the pre-existing models investigated, the Log-Transformed model appeared to be most reliable for the prediction of drug distribution into milk for basic drugs, however the prediction of acidic drugs was very variable and there is no formula for predicting neutral medications. We subsequently investigated whether interrogation of the formulae underpinning the Phase Distribution model could improve predictability. An alternative model for prediction of milk lipid distribution (Bartels-LogD model) improved predictions, but the use of observed *fu*_*m*_ or milk pH values; alternative equations for predicting *fu*_*m*_; or incorporation of ionisation into milk protein binding predictions failed to significantly improve model reliability. OCT1 substrates and drugs with a low *fu*_*p*_ were identified as more likely to be underpredicted.

The limited predictability of the Log-Transformed model has been highlighted previously, where analysis of 69 medications, assuming a milk pH of 7.2 and a Crt of 4.5%, showed no correlation between the predicted and observed M/P values [R^2^ = 0.01; (Larsen et al., 2003))] and this concern has also been raised by other groups (Yang et al., 2022; Pansari et al., 2024). Although the LogP_o:w_ model showed reasonable predictability compared with the other models, the use of LogP rather than the partition coefficient (i.e., the exponent of LogP; see Equations 4, 5) results in the model failing to recapitulate how changes in Crt affect drug distribution. For example, the LogP_o:w_ model predicts the M/P ratio of the lipophilic compound labetalol to increase from 0.80 to 0.91 as Crt increases from 3% to 12%, whereas the Phase Distribution model predicts this to rise from 2.95 to 9.50. As such, focusing on the non-log-transformed Phase Distribution model to derive better predictions was deemed most appropriate. This is supported by the knowledge that the LogP_o:w_ model is in fact a misrepresentation of the original Phase Distribution model [as detailed in (Zhang et al., 2022)], and that the authors have subsequently employed the Phase Distribution and Log-Transformed models (Pan et al., 2023; Abduljalil et al., 2022; Pansari et al., 2022). The ABI and Bartels-LogD models predict a significantly lower *Papp*_*milk*_ for drugs with a LogD of more than 3 (see Supplementary Figure S2), and the improved predictions of lipophilic drugs using these models demonstrate that the linear extrapolation of the Atkinson/Begg formula included in the Phase Distribution model may not be appropriate.

Our data showed that OCT1 substrates were significantly underpredicted, and this is consistent with a role for OCT1 in the active transport of drugs into the breastmilk. To the best of our knowledge, there has been little study into this role of OCT1 during lactation in humans, but rodent models have shown a reduction in the OCT1 substrate, thiamin, in the milk of OCT1 knockout mice (Kato et al., 2015). In support of this, OCT1 is upregulated during lactation (Kimura et al., 2006; Alcorn et al., 2002). Similarly, BCRP has been shown to be upregulated during lactation (Ahmadzai et al., 2022; Sychterz et al., 2024), and it was therefore surprising to see that the prediction reliability for BCRP substrates was not significantly different to that of drugs not mediated by transporters (Figure 4A; p = 0.068). We acknowledge that this may have been the result of using a limited dataset only, with other groups previously highlighting that BCRP substrates are likely to be actively transported into the breast milk (Ito et al., 2015; Yang et al., 2022; Sychterz et al., 2024; Gong et al., 2024). Similar to the present study, these previous works typically rely on a small number of BCRP substrates only. Prior work has demonstrated that *in vitro* to *in vivo* extrapolation (IVIVE) can be used to better predict the M/P ratio of BCRP substrates (Ito et al., 2015; Yang et al., 2022). This was considered outside the scope of the current work but is certainly an area in which more work should be focused, and gathering data from more substrates of BCRP (and other transporters) would clearly be beneficial. At present, these data are challenging to interpret given that the majority of medications are substrates for multiple transporters that are expressed within the breast. Our data agree well with the previously suggestion that MDR1 and MRP2 do not play a significant role in the transfer of drug into the breast milk (Alcorn et al., 2002; Ahmadzai et al., 2022; Gong et al., 2024).

From a clinical perspective, the underprediction of M/P ratios may contribute towards dosing nursing infants with unsafe quantities of maternal medications via the breastmilk, and therefore understanding the role of transporters such as BCRP and OCT1 is imperative. When considering only drugs that are not OCT1/BCRP substrates, 97% of medications were predicted within 2-fold of the observed data or overpredicted (2/64 underpredicted) when using the pH range of 6.63–7.62. Although overpredictions are not desirable *per se*, these scenarios allow the use of a model-based approach for estimating worst-case scenarios in the presence of limited clinical data. Whilst work should be encouraged to continue improving the reliability of such models, understanding of current limitations to ensure models are employed in appropriate ways is also beneficial.

It was somewhat surprising to see that using observed *fu*_*m*_ values and alternative models for predicting *fu*_*m*_ did not improve or even significantly affect predictions (Figure 3). The model assumes that only the unbound fraction of a drug is able to partition into milk lipids, and so increasing the milk protein binding will lead to a ‘compensatory’ increase in lipid partitioning, and therefore little change in predicted M/P ratio. Although this physiological mechanism supports our findings, there is little-to-no experimental evidence to verify the interplay between protein binding, lipid partitioning and ionisation. Atkinson and Begg’s original protein binding (Atkinson and Begg, 1988a) and lipid partitioning (Atkinson and Begg, 1988b) experiments were all performed at pH 7.2, as were more recent, similar, experiments (Ito et al., 2013). More detailed *in vitro* work is therefore needed to quantify the relationship between these factors in order to develop more reliable models.

The limited availability of paired milk pH, Crt, protein binding and M/P ratio data provides additional complications. The models can be used to predict the effect of variable milk physiology on drug accumulation, and therefore infant exposure, however there are little data available to verify this predicted effect of Crt or pH. Our publication list contained only two studies with an AUC M/P ratio and Crt data (Rampono et al., 2000; Kristensen et al., 2007). In one of these, fore- and hindmilk were analysed separately and shown to have Crt values of 6.2% and 13.7% respectively, which correlated with a 2.3-fold increase in mirtazapine concentration [LogP 2.9; (Kristensen et al., 2007)]. This change is recapitulated by both the Phase Distribution model (2.1-fold increase) and the Bartels-LogD model (1.89-fold increase), with the Bartels-LogD model more accurately predicting the M/P ratio (P/O ratio of 0.87–1.65 vs. 3.10–6.56; all at pH 7.12). Such a difference in the Crt of fore- and hindmilk is well established throughout the literature, with hindmilk typically exhibiting Crt values 2-3 times higher than that of the foremilk (Meier et al., 2006; Kristensen et al., 2007; Daly et al., 1993; Bowornkitiwong et al., 2023; Mizuno et al., 2009). These data may be beneficial when considering study design: if a drug has a low LogD at breast milk pH then it can be anticipated that samples of foremilk only will reflect drug concentrations in the whole milk. In contrast, for lipophilic drugs, sampling of whole milk or fore- and hindmilk will be considered more important. When we have a robust understanding of how changes in Crt affect dug distribution, it is plausible that modelling may be used to extrapolate whole milk values from fore-milk only, which may reduce the burden on women recruited to clinical trials. As stated, additional observed data with paired milk pH and Crt information are required to verify this suggestion, and will thus support the use of modelling in the optimisation of trial design. Variation of the milk pH and across postpartum time has also been reported and may affect drug distribution (Morriss et al., 1986; Ansell et al., 1977; Matheson et al., 1990), however the intra-individual and inter-individual variability likely outweighs the variability caused by the maturation of breastmilk production (see Supplementary Figure S4).

Breastmilk pH data were available in 15 publications, however this was presented as paired in only half of these (See Supplementary Table S2). Full interpretation of these data is challenging however, when the other parameters which are predicted to affect the M/P ratio are not simultaneously presented. Figure 6 showed that for a number of drugs, the predicted range of M/P ratios was >30-fold, however this is much larger than the observed range of M/P ratios. It is unclear whether this mismatch is due to our incomplete understanding of the effect of pH on M/P ratios (as discussed above), or because the range of milk pH’s collected in each study was more narrow than the physiological range simulated. It is acknowledged that studies quantifying all these milk parameters present a considerable technical challenge, particularly given that milk pH changes with storage time and this likely has knock-on effects for protein binding and lipase activity, which subsequently affects Crt and lipid partitioning (Erickson et al., 2013; Van Den Berg, 1961; Vázquez-Román et al., 2018). It should, however, be feasible to measure milk pH and Crt in milk aliquots prior to storage in order to pair these data with drug concentrations at a later timepoint.

In addition to the *quantity* of robust data available, another limiting factor in model development may be the *quality* of data available. To increase the reliability of observed M/P ratios only medications with milk AUC were included, however concerns do still exist. Many of the studies included within the datasets used were conducted over 30 years ago, at a time when analytical methods were less reliable than they are now. The development of deuterated internal standards has significantly improved our ability to quantify drug concentrations within the breastmilk, and our understanding and awareness of drug retention in labware has increased greatly (Alshogran et al., 2024; Lopes et al., 2016). With that in mind, it is noteworthy that the Bartels-LogD model predicted 17/18 non-transporter-mediated drugs within 2-fold of the observed data (across a pH range of 6.63–7.62) when considering publications from 1990 onwards (100% within 3-fold). In contrast, 10/24 drugs published prior to 1990 were mis-predicted, with 9 of these being over predictions. It is plausible to suggest that overpredictions may be more likely to occur due to the reduced reliability of drug extraction and the higher likelihood of sample degradation in older studies. As more clinical data collected in the modern era become available, it is likely that more reliable models may be developed.

Koshimichi et al. developed a semi-mechanistic model for the prediction of M/P ratios using linear regressions to predict the effect of physicochemical properties on secretion (CL_sec_) and reuptake (CL_re_) into the breastmilk (Koshimichi et al., 2011), with this model being incorporated into the PKsim software to predict infant exposure to 10 medications (80% were predicted within 2-fold; (Nauwelaerts et al., 2023)). Separate prediction of CL_sec_ and CL_re_ results in time-dependent differences in the predicted M/P ratio, thus offering an advantage over the models included in the present analysis which calculate a steady state M/P ratio only. The semi-mechanistic model predicted the M/P ratio of 72% of drugs within 3-fold of the observed data, compared with 71%–84% for the Bartels-LogD model (from Figure 6). More recently, an IVIVE model has been developed which incorporates an optimised efflux ratio to improve prediction of transport-mediated drugs (Yang et al., 2022). Across a dataset of 162 drugs (48 of were mediated by passive diffusion only), this model was shown to outperform other models with 66% of 162 drugs being predicted within 2-fold of the observed data compared with 51%, 44% and 43% for the Phase Distribution, Log-Transformed and Koshimichi models, respectively (Yang et al., 2022). This compares with 51%–72% for the Bartels-LogD model.

Overall, these data suggest that the Bartels-LogD model presented here may offer an improvement on existing mathematical models for predicting steady state M/P ratios and performs comparably to other, more mechanistic models published recently. The range of predicted milk-to-plasma ratios presented in this study for each drug demonstrates that consideration of the biological heterogeneity of breastmilk is important for model development and validation. The paucity of data supporting the physiological mechanisms underlying lipid partitioning, protein binding and the effect of ionisation therefore limits the development of models. Although there are technical challenges associated with clinical lactation studies which have hindered this, it is expected that increasing advances in bioanalysis and continuous effort to improve maternal and infant safety will lead to further improvements in model development.

## Data Availability

The original contributions presented in the study are included in the article/Supplementary Material, further inquiries can be directed to the corresponding author.
