# Supplementary material for: Evaluation of mathematical models for predicting medicine distribution into breastmilk - considering biological heterogeneity
Source: Front Pharmacol. 2024 Nov 29;15:1507551. doi: 10.3389/fphar.2024.1507551 (PMC11645658; doi:10.3389/fphar.2024.1507551)
Supplement: Supplementary file 1 [file DataSheet1.PDF]

Supplementary Material

1 Milk pH

Milk pH was extracted from 15 studies as listed in Table S2. Studies were weighted according to the number of women included in each study and a mean (± SD) pH of 7.12 ± 0.24 was calculated. This range was used for all simulations unless otherwise stated. Sources of these data can be found within Table S2.

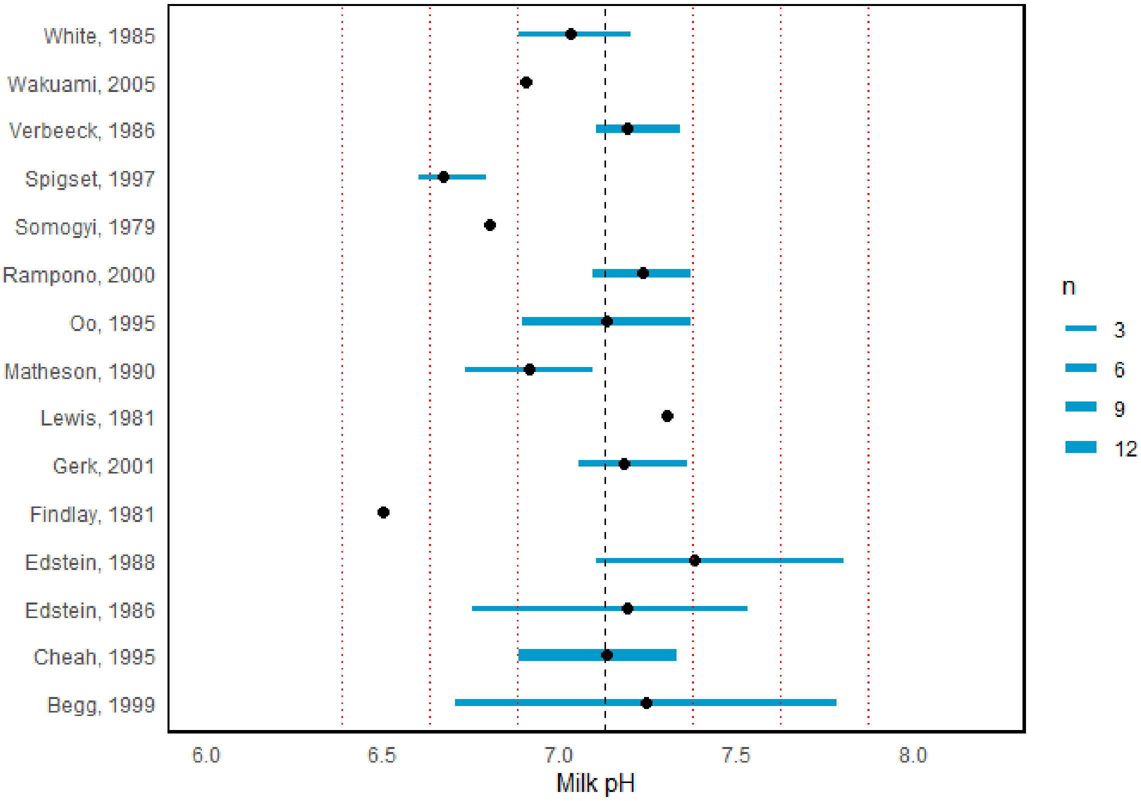

Figure S1: Mean (black dots) and range (blue bars) milk pH recorded from 15 studies. The weighted mean (7.12) is shown by the black dashed line, with markers for one (6.88 – 7.37), two (6.63 - 7.62) and three (6.38 – 7.87) standard deviations shown in red.

2 Alternative predictions of $Papp_{milk}$

Through evaluation of the literature, four new models for prediction of $Papp_{milk}$ were devised and evaluated. The original formulation for predicting $Papp_{milk}$ utilised *in vitro* experiments and drew a correlation between $LogD_{o:w}$ and partitioning between the aqueous and lipid phase of milk. This work evaluated 16 drugs, with two being excluded from the final fitting due to concerns regarding interactions with free fatty acids in the milk. All work was conducted at pH 7.2 and all drugs had a $LogD_{7.2}$ of between -0.5 and 3 [1].

2.1 ABI model

More recently, another group has performed similar experiments and characterised milk lipid partitioning for a wider number of drugs, with $LogD_{7.2}$ ranging from -2 to 5 [2]. We therefore pooled these data with those produced in the initial work to draw a new correlation between LogD and milk lipid partitioning. The pooled data were best fit with a linear equation in the form:

$$Papp_{milk} = 10^{(0.4017 \cdot LogD_{pH,milk}) + 0.1548}$$

Compared with the original model, the ABI model predicts that $Papp_{milk}$ will be lower for lipophilic drugs (See Figure S2).

2.2 MCDB model

Although relatively limited data are available regarding lipid partitioning in human milk, animal studies offer richer datasets. The Milk Composition Database (MCDB) provides quantitative on the creamatocrit (Crt) and drug concentration of a diverse spectrum of compounds in cow's milk [3]. Milk lipid partitioning was calculated for compounds with a $LogP_{o:w}$ between -5 and 10 as

$$Papp_{milk} = \frac{C_{lipid}}{C_{skim}}$$

Where:

$$C_{lipid} = \frac{C_{whole} - (C_{skim} \cdot (1 - Crt))}{Crt}$$

Where C_{whole}, C_{lipid} and C_{skim} are the concentration of drug in whole, lipid and skimmed milk respectively. C_{whole} and C_{skim} are provided within the MCDB. 679 datapoints were best fit by a one phase decay equation in the form:

$$Papp_{milk} = 10^{(2.162 - 5.327) \cdot \exp(-0.1153 \cdot LogP) + 5.327}$$

Compared with the original model, the MCDB model predicts reduced lipid partitioning for drugs with a LogP > 3, and increased lipid partitioning for drugs with a LogP < 3

2.3 Bartels models

Bartels et al published correlations between $LogP_{o:w}$ and the olive oil-to-water partition coefficient ($LogP_{vo:w}$) for drugs with a $LogP_{o:w}$ ranging from -2 to 8 [4]. This was considered of interest for the present work given that $LogP_{vo:w}$ may be a better predictor for partitioning into fatty tissues than $LogP_{o:w}$ due to the prevalence of triglycerides in both adipose tissue and olive oil, and the knowledge that breastmilk lipid predominantly consists of triglycerides [5-7]. A sigmoidal relationship between the two was identified, described by an equation in the form:

$$Log(Papp_{milk}) = -4.653 + \frac{7.972}{1 + 10^{0.1175-0.2849 \cdot LogP}}$$

Of particular interest (due to the results of lipophilic drugs presented in Figure 1) is that for drugs with a LogP < 3 the equation agrees well with that presented by Atkinson and Begg [1], with plateauing of the curve occurring at higher LogP values. We noted that this equation does not factor for the notion that ionised species are not predicted to enter the lipid phase, and so we also proposed and investigated the reliability of this equation using LogD instead of LogP:

$$Log(Papp_{milk}) = -4.653 + \frac{7.972}{1 + 10^{0.1175-0.2849 \cdot LogD_{pH,milk}}}$$

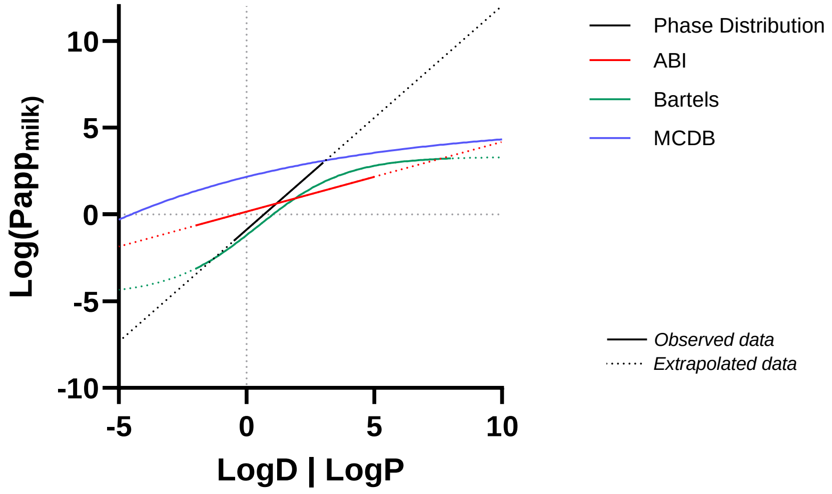

Figure S2: Relationship between $Papp_{milk}$ and LogD (Phase Distribution, ABI and Bartels-LogD models) or LogP (MCDB and Bartels-LogP models). The solid lines represent the range across which the equation was verified, with the dotted lines representing extrapolation of these equations.

3 Alternative formulations of fu_{milk}

The Yang model for predicting fu_m is the multiple Emax model presented previously and utilises polar surface area in addition to fu_p in the prediction of fu_m [8]:

$$fu_m = 1.033 \times fu_p/(0.988 + fu_p) + 0.1017 \times ln(PSA)$$

Ito et al [2] published measured values for fraction unbound in the milk, generated by experiments similar to those conducted by Atkinson and Begg in the development of Equation 2 [9]. As such, data from both groups were pooled, and these were best fit by a linear equation in the form:

$$fu_m = \left(fu_p \times 0.4956\right) + 0.5335$$

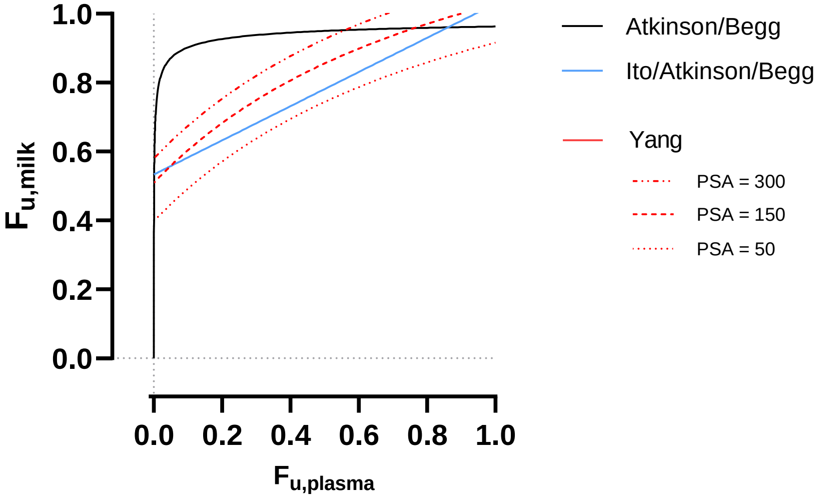

Figure S3: Relationship between the prediction fu_m and observed fu_p values for the Atkinson/Begg (black), Ito/Atkinson/Begg (blue) and Yang (red) models.

The effect of ionisation on milk protein binding was assumed to be proportional to the effect on binding to plasma proteins [10]. Predicted *fu* at the relevant pH was calculated as the average of fu_p for the uncharged (neutral), positively charged (basic), and negatively charged (acidic) species respectively, weighted by the fraction charged/uncharged that was present at the relevant pH:

$$fu_{pH,milk} = \left(\frac{1}{1+\ 10^{0.4485LogP-0.4782}} \times f_{uncharged}\right)$$
$$+\left(\frac{1}{1+\ 10^{0.4628LogP-1.0971}} \times f_{positive}\right)$$
$$+\left(\frac{1}{1+\ 10^{0.3649LogP+0.4162}} \times f_{negative}\right)$$

Proportional change in the bound fraction was then calculated through normalisation to the predicted protein binding at pH 7.4:

$$Proportional\ Change\ in\ Bound\ Fraction\ (PCBF) = \frac{fu_{pH,milk}}{fu_{pH7.4}}$$

This proportional change was applied to the Atkinson/Begg, Yang and Ito predicted values for fu_m:

$$fu_{m,ionised} = fu_{m,unionised} \times PCBF$$

4 Postpartum changes in milk properties

Previous reports have demonstrated that colostrum and mature milk exhibit distinct physiological profiles to match the needs of the developing infant. It was considered that this may be associated with changes in pH and/or creamatocrit, which could lead to changes in drug distribution as postpartum time progresses. Literature was therefore reviewed to investigate the value of modelling distinct 'types' of milk. Data were extracted from >1200 milk samples, weighted for sample size and fit with linear fittings across the first postpartum year (pH & Crt) and across the first postpartum fortnight only (pH only, due to lack of Crt data) in order to investigate compositional changes in mature milk and colostrum respectively.

Mean milk pH was ~7.1 (Fig. S4A) and milk Crt was ~5% (Fig. S4B). There was no significant change in milk pH (slope = 0.0001) or Crt (slope = -0.002) across the first postpartum year. When evaluating colostrum only, the slope of the line was -0.025, representing a decline in pH from ~7.15 to ~6.95 across the first two postpartum weeks. We did not fit the colostrum Crt data as only three datapoints were identified. These data points were 4.45 ± 1.75% (day 0), 6.53 ± 3.83% (d3) and 6.61 ± 1.82% (d7).

Overall, these data suggest that while changes in breastmilk composition may occur across postpartum time, the intra-individual and inter-individual variability significantly outweigh the variability caused by the maturation of breastmilk production.

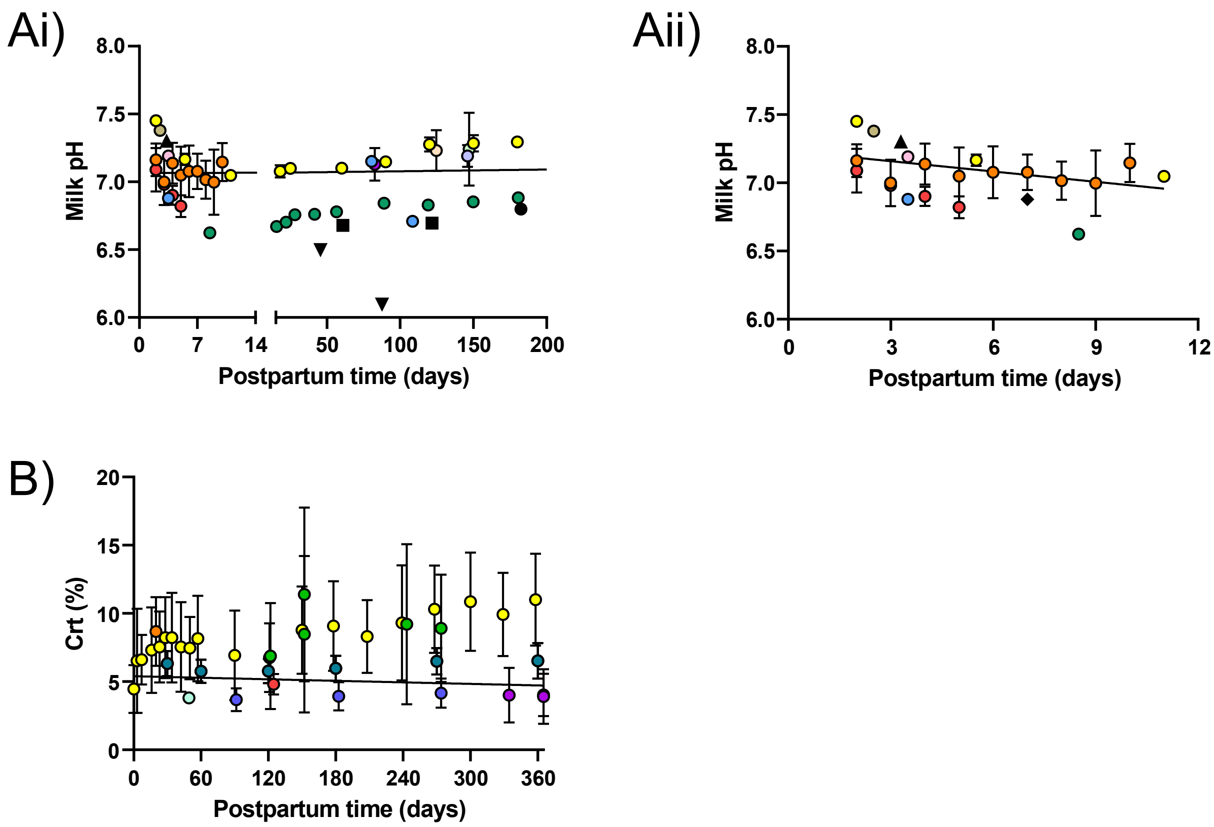

Figure S4: Changes in milk pH (A) and Crt (B) across postpartum time. Linear fittings were weighted for sample size.

Data in A were collated from Oo et al (1995; purple [11]) ; Somogyi et al (1979; black circle [12]); Spigset et al (1997; black square [13]); Rampono et al (2000; light orange [14]); Lewis et al (1981; black triangle [15]); Matheson et al (1990, red [16]); Begg et al (1999; lime green [17]); Verbeeck et al (1988; light purple [18]); Edstein et al (1986; pink [19]); Ansel et al (1977; orange [20]); Allen et al (1991; dark green [21]); Malhotra et al (1982; light blue [22]); White et al (1985; black diamond [23]); Findlay et al (1981; black triangle, downwards [24]); Edstein et al (1988; gold [25]) and Morriss et al (1986; yellow [26]).

Data in B were collated from Rampono et al (2000; red [14]); Meier et al (2005; orange [27]); Allen et al (1991; yellow [21]); Daly et al (1993; green [28]); Butts et al (2018; pale green [29]); Mitoulas et al (2002; turquoise [30]); Arcus-Arth et al (2005; blue [31]) and Perrin et al (2016; purple [32]).

5 Supplementary references

1. Atkinson, H.C. and E.J. Begg, *Relationship between human milk lipid-ultrafiltrate and octanol-water partition coefficients.* J Pharm Sci, 1988. **77**(9): p. 796-8.
2. Ito, N., et al., *Contribution of protein binding, lipid partitioning, and asymmetrical transport to drug transfer into milk in mouse versus human.* Pharm Res, 2013. **30**(9): p. 2410-22.
3. Foroutan, A., et al., *Chemical Composition of Commercial Cow's Milk.* J Agric Food Chem, 2019. **67**(17): p. 4897-4914.
4. Bartels, M., et al., *Development of PK- and PBPK-based modeling tools for derivation of biomonitoring guidance values.* Comput Methods Programs Biomed, 2012. **108**(2): p. 773-88.
5. Poulin, P., K. Schoenlein, and F.P. Theil, *Prediction of adipose tissue: plasma partition coefficients for structurally unrelated drugs.* J Pharm Sci, 2001. **90**(4): p. 436-47.
6. Chamberlin, A.C., et al., *Modeling free energies of solvation in olive oil.* Mol Pharm, 2008. **5**(6): p. 1064-79.
7. Ramiro-Cortijo, D., et al., *Breast Milk Lipids and Fatty Acids in Regulating Neonatal Intestinal Development and Protecting against Intestinal Injury.* Nutrients, 2020. **12**(2).
8. Yang, H., et al., *Developing an In Vitro to In Vivo Extrapolation (IVIVE) Model to Predict Human Milk-to-Plasma Drug Concentration Ratios.* Mol Pharm, 2022. **19**(7): p. 2506-2517.
9. Atkinson, H.C. and E.J. Begg, *Prediction of drug concentrations in human skim milk from plasma protein binding and acid-base characteristics.* Br J Clin Pharmacol, 1988. **25**(4): p. 495-503.
10. Lobell, M. and V. Sivarajah, *In silico prediction of aqueous solubility, human plasma protein binding and volume of distribution of compounds from calculated pKa and AlogP98 values.* Mol Divers, 2003. **7**(1): p. 69-87.
11. Oo, C.Y., et al., *Pharmacokinetics in lactating women: prediction of alprazolam transfer into milk.* Br J Clin Pharmacol, 1995. **40**(3): p. 231-6.
12. Somogyi, A. and R. Gugler, *Cimetidine excretion into breast milk.* Br J Clin Pharmacol, 1979. **7**(6): p. 627-9.
13. Spigset, O., et al., *Excretion of citalopram in breast milk.* Br J Clin Pharmacol, 1997. **44**(3): p. 295-8.
14. Rampono, J., et al., *Citalopram and demethylcitalopram in human milk; distribution, excretion and effects in breast fed infants.* British Journal of Clinical Pharmacology, 2000. **50**(3): p. 263-268.
15. Lewis, A.M., et al., *Mexiletine in human blood and breast milk.* Postgrad Med J, 1981. **57**(671): p. 546-7.
16. Matheson, I., P.K. Lunde, and J.E. Bredesen, *Midazolam and nitrazepam in the maternity ward: milk concentrations and clinical effects.* Br J Clin Pharmacol, 1990. **30**(6): p. 787-93.
17. Begg, E.J., et al., *Paroxetine in human milk.* Br J Clin Pharmacol, 1999. **48**(2): p. 142-7.
18. Verbeeck, R.K., S.G. Ross, and E.A. McKenna, *Excretion of trazodone in breast milk.* Br J Clin Pharmacol, 1986. **22**(3): p. 367-70.

19. Edstein, M.D., et al., *Excretion of chloroquine, dapsone and pyrimethamine in human milk.* Br J Clin Pharmacol, 1986. **22**(6): p. 733-5.

20. Ansell, C., A. Moore, and H. Barrie, *Electrolyte pH changes in Human Milk.* Pediatr Res, 1977. **11**(12): p. 1177-9.

21. Allen, J.C., et al., *Studies in human lactation: milk composition and daily secretion rates of macronutrients in the first year of lactation.* Am J Clin Nutr, 1991. **54**(1): p. 69-80.

22. Malhotra, S.L., *Effect of non-suckling on the pH of breast milk and its possible relationship with breast cancer.* Postgrad Med J, 1982. **58**(686): p. 749-52.

23. White, W.B., J.W. Andreoli, and R.D. Cohn, *Alpha-methyldopa disposition in mothers with hypertension and in their breast-fed infants.* Clin Pharmacol Ther, 1985. **37**(4): p. 387-90.

24. Findlay, J.W., et al., *Analgesic drugs in breast milk and plasma.* Clin Pharmacol Ther, 1981. **29**(5): p. 625-33.

25. Edstein, M.D., J.R. Veenendaal, and R. Hyslop, *Excretion of mefloquine in human breast milk.* Chemotherapy, 1988. **34**(3): p. 165-9.

26. Morriss, F.H., Jr., et al., *Relationship of human milk pH during course of lactation to concentrations of citrate and fatty acids.* Pediatrics, 1986. **78**(3): p. 458-64.

27. Meier, P.P., et al., *Accuracy of a user-friendly centrifuge for measuring creamatocrits on mothers' milk in the clinical setting.* Breastfeed Med, 2006. **1**(2): p. 79-87.

28. Daly, S.E., et al., *Degree of breast emptying explains changes in the fat content, but not fatty acid composition, of human milk.* Exp Physiol, 1993. **78**(6): p. 741-55.

29. Butts, C.A., et al., *Human Milk Composition and Dietary Intakes of Breastfeeding Women of Different Ethnicity from the Manawatu-Wanganui Region of New Zealand.* Nutrients, 2018. **10**(9).

30. Mitoulas, L.R., et al., *Variation in fat, lactose and protein in human milk over 24 h and throughout the first year of lactation.* Br J Nutr, 2002. **88**(1): p. 29-37.

31. Arcus-Arth, A., G. Krowech, and L. Zeise, *Breast milk and lipid intake distributions for assessing cumulative exposure and risk.* J Expo Anal Environ Epidemiol, 2005. **15**(4): p. 357-65.

32. Perrin, M.T., et al., *A longitudinal study of human milk composition in the second year postpartum: implications for human milk banking.* Matern Child Nutr, 2017. **13**(1).
